# Elliptic flow and $R_{AA}$ of $\text {D}$ mesons at FAIR comparing the UrQMD hybrid model and the coarse-graining approach

**DOI:** 10.1140/epjc/s10052-019-6537-6

**Published:** 2019-01-21

**Authors:** Gabriele Inghirami, Hendrik van Hees, Stephan Endres, Juan M. Torres-Rincon, Marcus Bleicher

**Affiliations:** 1 Frankfurt Institute for Advanced Studies (FIAS), Ruth-Moufang-Str. 1, 60438 Frankfurt am Main, Germany; 2 Institut für Theoretische Physik, Johann Wolfgang Goethe-Universität, Max-von-Laue-Str. 1, 60438 Frankfurt am Main, Germany; 3 GSI Helmholtzzentrum für Schwerionenforschung GmbH, Planckstraße 1, 64291 Darmstadt, Germany; 4 John von Neumann Institute for Computing, Forschungszentrum Jülich, 52425 Jülich, Germany; 5 Present Address: Department of Physics, University of Jyvaskyla, P.O. Box 35, FI-40014 Jyvaskyla, Finland; 6 Department of Physics and Astronomy, Stony Brook University, Stony Brook, NY 11794 USA

## Abstract

We present a study of the elliptic flow and $R_{AA}$ of $\text {D}$ and $\bar{\text {D}}$ mesons in Au+Au collisions at FAIR energies. We propagate the charm quarks and the $\text {D}$ mesons following a previously applied Langevin dynamics. The evolution of the background medium is modeled in two different ways: (I) we use the UrQMD hydrodynamics + Boltzmann transport hybrid approach including a phase transition to QGP and (II) with the coarse-graining approach employing also an equation of state with QGP. The latter approach has previously been used to describe di-lepton data at various energies very successfully. This comparison allows us to explore the effects of partial thermalization and viscous effects on the charm propagation. We explore the centrality dependencies of the collisions, the variation of the decoupling temperature and various hadronization parameters. We find that the initial partonic phase is responsible for the creation of most of the $\text {D}/\bar{\text {D}}$ mesons elliptic flow and that the subsequent hadronic interactions seem to play only a minor role. This indicates that $\text {D}/\bar{\text {D}}$ mesons elliptic flow is a smoking gun for a partonic phase at FAIR energies. However, the results suggest that the magnitude and the details of the elliptic flow strongly depend on the dynamics of the medium and on the hadronization procedure, which is related to the medium properties as well. Therefore, even at FAIR energies the charm quark might constitute a very useful tool to probe the quark–gluon plasma and investigate its physics.

## Introduction

Heavy quarks represent an excellent method to probe the hot and dense medium which is supposed to form in heavy ion collisions [1]. Their mass $M_{\text {HF}}$ is much larger than $\Lambda _{\text {QCD}}$ and $T_{\text {QGP}}$, therefore we can use perturbative QCD (pQCD) [2] to model their production as a hard process [3] which happens mostly during the initial collision processes and almost negligibly by thermal production, except at early times at LHC energies [4]. Once formed, since the strong interaction conserves the flavour quantum number, the heavy quarks maintain their identity until the hadrons they form decay by weak (or in the case of the $\text {J}/\Psi$ by electro-magnetic) interaction. Moreover, since the energy loss in the medium due to multiple scattering and induced gluon bremsstrahlung depends on the mass of the propagating particle [5–7], heavy quarks are less affected than light quarks by the interactions with the medium and they convey information about the whole system evolution. At high transverse momenta the interest is oriented toward studying the opacity of the medium through the particle suppression in the high $p_{\text {T}}$ range, as observed in the experimental nuclear modification factor [8, 9]. In the low-$p_{\text {T}}$ range the focus is on in-medium hadronization and thermalization [10], reached by charm quarks at LHC energies, as theoretical considerations suggest [11–13] and as the observed experimental elliptic flow proves [14]. Numerical simulations, which are an essential tool to connect theory with experiments, are continuously improved to provide a consistent, realistic description of the heavy-quark propagation [15], adopting many different approaches [16–19] and investigating also small systems [20].

In this paper we study the elliptic flow and the $R_{AA}$^1^ of $\text {D}$ and $\bar{\text {D}}$ mesons in Au+Au collisions at $\sqrt{s_{NN}}\,\simeq 7 \, \text {GeV}$, a collision energy in the range of the upcoming FAIR facility [21], but also available at RHIC, within the Beam Energy scan program [22], and at NICA [23]. We adopt a Langevin propagation model, implicitly assuming that the heavy quark momentum transfer is much smaller than for the light partons, an approximation that at low collision energies should work reasonably well, while at RHIC and LHC energies it is really consistent only for bottom quarks [24]. After a brief introduction to the bulk evolution models that we use, i.e. the UrQMD hybrid model [25–27] and the coarse graining approach [28], we shortly review the formalism of the relativistic Langevin propagation, then we provide a basic overview of how we compute the transport coefficients, both for charm quarks and $\text {D}$ mesons. After showing and commenting the results of the simulations, we discuss how we might improve them.

## Models of the medium bulk evolution

### The UrQMD hybrid model

The primary bulk evolution of the medium is simulated using the hydrodynamics + Boltzmann setup the UrQMD hybrid model [25–27], adopting fluctuating initial conditions [29]. In the initial stage, UrQMD follows the elastic and inelastic collisions between nucleons, including color-flux-tube excitation and fragmentation processes. The hydrodynamical phase starts when the two Lorentz contracted nuclei have completely passed through each other, at $t=(2R)/(\sqrt{\gamma ^2_{\text {CM}}-1})$, where *R* is the radius of the two nuclei and $\gamma _{\text {CM}}$ is their Lorentz $\gamma$ factor in the center of mass frame [30]. The initial momentum, energy and baryon density distributions are created by summing the individual particle distributions, assumed to be three-dimensional Gaussians, like, e.g. for the energy density [31, 32]:

1 $$\begin{aligned} \varepsilon _{x,y,z}= & {} \left( \dfrac{1}{2\pi }\right) ^{\frac{3}{2}}\dfrac{\gamma _z E_p}{\sigma ^3} \nonumber \\&\times \exp \left[ -\frac{(x\!-\!x_p)^2\!+\!(y-y_p)^2+\gamma _z^2(z-z_p)^2}{2\sigma ^2} \right] ,\nonumber \\ \end{aligned}$$

where $E_p$ and $x_p,y_p,z_p$ are the energy and the coordinates of the particle in the computational frame, $\sigma$ is the width of the Gaussian (by default, $1\,\text {fm}$) and $\gamma _z$ is the Lorentz $\gamma$ factor to take into account the Lorentz contraction in the beam direction. UrQMD computes the fluid evolution by solving the differential equations which describe the conservation of total energy and net-baryon number, i.e.,

2 $$\begin{aligned} \partial _{\mu } T^{\mu \nu }=0, \quad \partial _{\mu }N^{\mu }=0, \end{aligned}$$

where $T^{\mu \nu }$ is the energy-momentum tensor and $N^{\mu }$ the baryon four-current. The hydro evolution is based on the SHASTA (SHarp And Smooth Transport Algorithm) algorithm [33, 34]; it exploits the chiral equation of state (EoS) [35] and assumes local thermal equilibrium, i.e. it does not take into account dissipative effects [36–40]. The hydrodynamical simulation is stopped when the maximum of the energy density on the grid becomes smaller than a certain value chosen for *particlization*. In the present work, we stopped the simulations at this point. Nevertheless in the full UrQMD hybrid model a rather advanced method to determine the freeze-out hypersurface is employed [41] and the energy density distribution is converted back to particles [42] through the Cooper–Frye [43] equation. Afterwards, the hadrons continue to scatter and strongly decay until no more interactions take place. To partially take into account this hadronic phase, we adopted a freeze-out temperature slightly below the standard value.

### The UrQMD coarse-graining approach

The hybrid model uses a microscopic description only for the very initial collisions and the final state interactions after the hydrodynamic phase, but it is also possible to extract macroscopic quantities from an underlying microscopic simulation during the whole collision evolution, as realized within the coarse-graining approach. It was first proposed in ref. [44] and has proven to account for the reaction dynamics and the production of electromagnetic probes successfully from SIS 18 to LHC energies [28, 45–48]. In this approach an ensemble of collision events simulated with a transport model (here: UrQMD in cascade mode) is put on a grid of small space-time cells. By averaging over a sufficient number of events the hadronic distribution function $f({{\varvec{x}}},{{\varvec{p}}},t)$ obtains a smooth form as

3 $$\begin{aligned} f({{\varvec{x}}},{{\varvec{p}}},t)=\left\langle \sum _{h} \delta ^{(3)}\left( {{\varvec{x}}}-{{\varvec{x}}}_{h}(t)\right) \delta ^{(3)}\left( {{\varvec{p}}}-{{\varvec{p}}}_{h}(t)\right) \right\rangle , \end{aligned}$$

where the angle brackets denote the ensemble average. It is then possible to extract the energy momentum tensor and the baryon current locally in space and time, i.e. for each cell of the grid. These quantities are given by the relations

4 $$\begin{aligned} T^{\mu \nu }({{\varvec{x}}},t)= & {} \frac{1}{\Delta V}\left\langle \sum \limits _{i=1}^{N_{h} \in \Delta V} \frac{p^{\mu }_{i} p^{\nu }_{i}}{p^{0}_{i}}\right\rangle , \end{aligned}$$

5 $$\begin{aligned} j^{\mu }_{\text {B}}({{\varvec{x}}},t)= & {} \frac{1}{{\Delta } V}\left\langle \sum \limits _{i=1}^{N_{\text {B}/\bar{\text {B}}} \in \Delta V}\pm \frac{p^{\mu }_{i}}{p^{0}_{i}}\right\rangle . \end{aligned}$$

Here $\Delta V$ denotes the cell volume and the sums are taken over the numbers of all hadrons $N_{h}$ or (anti-)baryons $N_{\text {B}/\bar{\text {B}}}$, respectively. In addition to the three components of the fluid velocity (using Eckart’s frame definition [49]) from $j^{\mu }$ in Eq. (5), the energy and the baryon densities in the cells can be obtained from the local rest-frame (LRF) values as

6 $$\begin{aligned} \varepsilon= & {} T^{00}_{\text {LRF}}, \end{aligned}$$

7 $$\begin{aligned} \rho _{\text {B}}= & {} j_{\text {B,\,LRF}}^{0}. \end{aligned}$$

Finally, by applying an EoS the local temperature *T* and baryon chemical potential $\mu _{\text {B}}$ are calculated from the energy density and the baryon density. For the present study a hadron gas EoS [50] with the same degrees of freedom as in the UrQMD transport model is applied, providing consistency with the underlying microscopic description which is purely hadronic. However, note that this may no longer be a fully valid picture, if the temperature in the fireball exceeds the critical temperature $T_{c}$, for which a phase transition to a quark–gluon plasma is expected. But because the maximum temperatures in collisions at FAIR energies are not found to be significantly above $T_{c}$, the differences compared to a full treatment of the phase transition by using an EoS [51, 52] fitted to lattice QCD results in the limit of $\mu _{B}=0$ are rather small (see comparison in Ref. [28]).

The determination of thermodynamic quantities for each cell via the coarse-graining approach requires – as in all macroscopic descriptions – the assumption of kinetic (and chemical) equilibrium, but in the underlying microscopic transport model these conditions are not always completely fulfilled. Therefore, deviations from the equilibrium state need to be considered. For the present case, the most relevant non-equilibrium effect shows up in the form of kinetic anisotropies, especially in the very early stages of the collision, due to the strong compression of the nuclei in longitudinal direction. Here, this non-equilibrium effect is eliminated by calculating the “effective”, i.e. thermalized, energy density using the framework given in Ref. [53].

For the sake of clarity, we stress that the UrQMD/coarse-graining approach allows the computation of the same physical quantities as in the UrQMD/hydro model, namely the three components of the fluid velocity, the energy density, the baryon density and, by introducing an EoS, also the temperature and the baryon chemical potential. Therefore the data coming from the UrQMD/coarse-graining approach can be used as a replacement of the UrQMD/hybrid-approach, providing an alternative description of the evolution of the medium based on transport models. However, there is an important difference in the utilization of the two approaches: while in the UrQMD/hybrid model we can perform the propagation of the heavy quarks and the computation of the medium dynamics at the same time, in the UrQMD/coarse-graining model the dynamical evolution of the background medium is calculated in advance by averaging many events and saved in a file, containing the fluid evolution data at fixed intervals of time. This means that, in the UrQMD/coarse-graining approach, the background medium evolution remains the same for all events in a certain centrality class. Nevertheless, we still have fluctuations in the final results due to the different initial positions and momenta of the heavy quarks, which vary event by event, and to their stochastic equations of motion. In a previous work [54] we found that the nuclear modification factor and the elliptic flow of *D* mesons seem to not change appreciably if, instead of averaging the final results of many events, we average the medium evolution, provided that the numerical sample of particles is the same and in the limit of the approximations adopted in our model, described in Sect. 5. Therefore, we consider our approach reasonable. For the present study, to compute the background medium evolution, we averaged $1.44\cdot 10^5$ events for reactions with impact parameter $b=3\,\text {fm}$ and $2.64\cdot 10^5$ events for reactions with $b=7\,\text {fm}$.

## The relativistic Langevin propagation of the charm quarks

Since the mass of the charm quarks is much larger than the mass of up, down and even strange quarks and since it is also much larger than the typical temperatures of the system, it is reasonable to assume that each collision with other particles will change the momenta of the charm quarks only by a small amount. Under these conditions, the Boltzmann equation can be approximated by a Fokker–Planck equation, which, in turn, can be recasted as an equivalent stochastic Langevin equation [10, 11, 55–60].

When dealing with relativistic speeds, we can formulate the Langevin process as:

8 $$\begin{aligned} \text {d}x_j= & {} \frac{p_j}{E} \text {d}t, \nonumber \\ \text {d}p_j= & {} -\Gamma p_j \text {d}t + \sqrt{\text {d}t} C_{jk} \rho _k. \end{aligned}$$

In Eq. (8) $E=\sqrt{m^2+\varvec{p}^2}$, $\text {d}t$ is the advancement time step, $\text {d}x_j$ and $\text {d}p_j$ are the variations of coordinates and momentum in each time-step, the $\rho _k$ are random variables distributed according to a normalized Gaussian distribution, $\Gamma$ and $C_{jk}$ are the drag or friction coefficient and the covariance matrix of the fluctuating force respectively, both defined in the local rest frame of the fluid and depending on $(t,\varvec{x},\varvec{p})$. These parameters of the Langevin process in Eq. (8) are related to the drag and diffusion coefficients *A*, $B_0$ and $B_1$ for an isotropic medium by

9 $$\begin{aligned} A p_j&=\Gamma p_j - \xi C_{lk} \frac{\partial C_{jk}}{\partial p_l}, \end{aligned}$$

10 $$\begin{aligned} C_{jk}&=\sqrt{2 B_0} P_{jk}^{\perp } + \sqrt{2 B_1} P_{jk}^{\parallel }, \end{aligned}$$

11 $$\begin{aligned} \text {with} \quad P_{jk}^{\parallel }&=\frac{p_j p_k}{\varvec{p}^2}, \quad P_{jk}^{\perp }=\delta _{jk}-\frac{p_j p_k}{\varvec{p}^2}. \end{aligned}$$

It is known that, modeling the medium in global thermal equilibrium, i.e. in a homogeneous static background medium, the stationary equilibrium limit should be a Boltzmann-Jüttner distribution,

12 $$\begin{aligned} f_Q^{(\text {eq})}(\varvec{p})=\exp \left( -\frac{E}{T} \right) . \end{aligned}$$

Therefore it is possible to tune the drag coefficient in Eq. (10) by choosing the longitudinal diffusion coefficient $B_1$ such as to satisfy this asymptotic equilibration condition [61], leading to dissipation-fluctuation relations between this diffusion coefficient and the drag coefficient [11, 62]. Essentially, if the dissipation-fluctuation relation,

13 $$\begin{aligned}&\Gamma (E) E T-D(E)+ T(1-\xi ) D'(E)=0, \nonumber \\&\quad \text {with}\, D(E)=B_1 \end{aligned}$$

is fulfilled, Eq. (12) becomes a solution of the corresponding stationary Fokker-Planck equation. In the post-point Ito realization [54, 63] $\xi =1$, and this choice allows to reduce Eq. (13) to

14 $$\begin{aligned} D(E)=\Gamma (E) E T, \end{aligned}$$

so that, after $\Gamma$ is computed from underlying microscopic models for heavy-quark scattering with light quarks and gluons introduced in the next section, the longitudinal diffusion coefficient $B_1$ is given by

15 $$\begin{aligned} B_1=\Gamma E T. \end{aligned}$$

We remind that in the derivation of the Langevin process we assumed to be in the rest frame of the background medium, therefore, when this procedure is applied in a dynamically evolving medium, it is necessary to first perform a boost to the comoving frame of the medium and then, after performing the Langevin propagation, to perform another boost back to the computational frame.

## Drag and diffusion coefficients

### Drag and diffusion coefficients for charm quarks

**Fig. 1:**
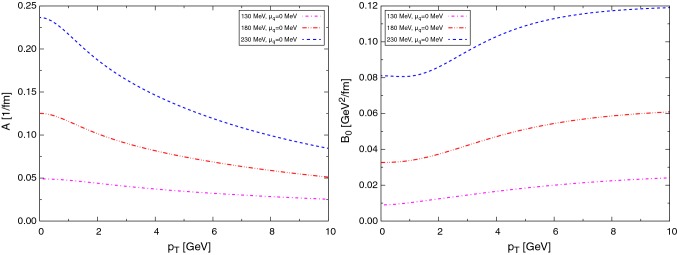
Drag (left) and diffusion (right) coefficients in the resonance model for charm quarks at different temperatures

**Fig. 2:**
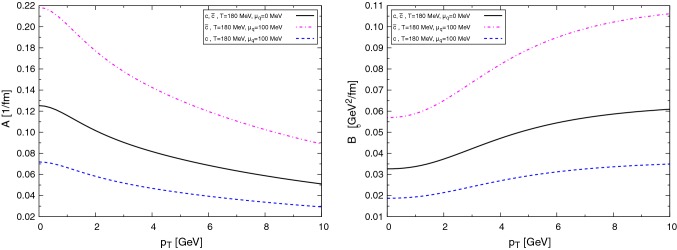
Drag (left) and diffusion (right) coefficients in the resonance model for charm quarks at different temperatures taking into account also a fugacity factor $\text {e}^{(-\mu /T)}$ for charm and $\text {e}^{(\mu /T)}$ for anti-charm quarks

In this work the drag and diffusion coefficients to perform the Langevin propagation of charm quarks are obtained from a resonance model, in which the existence of $\text {D}$ mesons in the QGP phase is assumed. The resonance model is based on heavy-quark effective theory (HQET) and chiral symmetry in the light-quark sector [57]. In this model we assume the existence of open-heavy-flavor meson resonances like the $\text {D}$ mesons, an assumption supported by the finding in lattice-QCD calculations that hadron-like bound states and/or resonances might survive the phase transition in both the light-quark sector (e.g., $\rho$ mesons) and heavy quarkonia (e.g., $\text {J}/\psi$) (Figs. 1, 2).

The heavy-light quark resonance model [57] is based on the Lagrangian:16where *v* is the heavy-quark four-velocity. The free part of the Lagrangian is given by17in which $\Phi$ and $\Phi _0^*$ are pseudo-scalar and scalar meson fields (corresponding to $\text {D}$ and $\text {D}_0^*$ mesons). Because of the chiral symmetry restoration in the QGP phase, the existence of mass degenerate chiral-partner states is also assumed. Further from heavy-quark effective symmetry it is expected to have spin independence for both the coupling constants, $G_S=G_V$, and the masses, $m_S=m_V$. For the strange-quark states we consider only the vector and pseudo-scalar states ($D_s^*$ and $D_s$, respectively).

The $\text {D}$-meson propagators are dressed with the corresponding one-loop self energy. Assuming charm-quark masses of $m_c=1.5\;\text {GeV}$, we adjust the masses of the physical $\text {D}$-meson-like resonances to $m_{\text {D}}=2 \; \text {GeV}$, in approximate agreement with the *T*-matrix models of heavy-light quark interactions in [64, 65]. The strong-coupling constant is chosen as $\alpha _s=g^2/(4 \pi )=0.4$, such as to obtain resonance widths of $\Gamma _{D} =0.75 \; \text {GeV}$.

We use these propagators to compute the elastic *Qq*- and $Q\overline{q}$-scattering matrix elements, which are then used in Eqs. (18) and (20) for the evaluation of the pertinent drag and diffusion coefficients for the heavy quarks. It turns out that particularly the *s*-channel processes through a $\text {D}$-meson like resonance provide a large efficiency for heavy-quark diffusion compared to the pQCD cross sections for the same elastic scattering processes, resulting in charm-quark equilibration times $\tau _{\text {eq}}^c =2-10 \; \text {fm}/c$.

The relation of elastic heavy-quark-scattering matrix elements with the drag and diffusion coefficients in the Langevin approach is given by integrals of the form

18 $$\begin{aligned} \left\langle {X(\varvec{p}')} \right\rangle= & {} \frac{1}{2 \omega _{\varvec{p}}} \nonumber \\&\times \int _{\mathbb {R}^3} \frac{\text {d}^{3} \varvec{q}}{2E(\varvec{q}) \, (2\pi )^{3}} \int _{\mathbb {R}^3} \frac{\text {d}^{3} \varvec{p}'}{2E(\varvec{p}') \, (2\pi )^{3}}\int _{\mathbb {R}^3} \frac{\text {d}^{3} \varvec{q}'}{2E(\varvec{q}') \, (2\pi )^{3}} \nonumber \\&\times \frac{1}{\gamma _{Q}} \sum _{g,q}|{\mathcal {M}}|^2 (2 \pi )^{4} \delta ^{(4)}(p+q-p'-q') f_{q,g}(\varvec{q}) X(\varvec{p}') \ ,\nonumber \\ \end{aligned}$$

where the invariant scattering-matrix elements are

19 $$\begin{aligned} \sum |\mathcal {M}|^2= & {} \frac{64 \pi }{s^2} (s-m_q^2+m_Q^2)^2(s-m_Q^2-m_q^2)^2 \nonumber \\&\times {N}_f \sum _{a} d_a \left( |T_{a,l=0}(s)|^2+ 3|T_{a,l=1}(s) \cos \theta _{\text {cm}}|^2 \right) .\nonumber \\ \end{aligned}$$

In Eq. (18) the integrations run over the three momenta of the incoming light quark or gluon and the momenta of the outgoing particles. The sum over the matrix element is taken over the spin and color degrees of freedom of both the incoming and outgoing particles; $\gamma _Q=6$ is the corresponding spin-color degeneracy factor for the incoming heavy quark, and $f_{q,g}$ stands for the Boltzmann distribution function for the incoming light quark or gluon. When adopting this notation, the drag and diffusion coefficients are given by

20 $$\begin{aligned} \begin{aligned} A(\varvec{p})&= \left\langle {1-\frac{\varvec{p} \varvec{p}'}{\varvec{p}^2}} \right\rangle , \\ B_{0}(\varvec{p})&= \frac{1}{4} \left\langle {\varvec{p}'{}^2-\frac{(\varvec{p}' \varvec{p})^2}{\varvec{p}^2}} \right\rangle , \\ B_{1}(\varvec{p})&= \frac{1}{2} \left\langle {\frac{(\varvec{p'} \varvec{p})^2}{\varvec{p}^2} - 2 \varvec{p}' \varvec{p} + \varvec{p}^2} \right\rangle . \end{aligned} \end{aligned}$$

We also include the leading-order perturbative QCD cross sections for elastic gluon heavy-quark scattering [66], including a Debye screening mass $m_{\text {D}g}=g T$ in the gluon propagators, thus controlling the *t*-channel singularities in the matrix elements.

### Drag and diffusion coefficients for D-mesons

To account for the combined effect of $\text {D}^+$ and $\text {D}^0$ ($\text {D}^-$ and $\bar{\text {D}}_0$) mesons we implement the transport coefficients using the $\text {D}$-meson ($\bar{\text {D}}$-meson) isospin-averaged scattering amplitudes. In this way we are incorporating possible “off-diagonal transitions” in which the heavy meson can exchange flavor like $\text {D}^+ \pi ^0 \rightarrow \text {D}^0 \pi ^+$.

Below the hadronization temperature the $\text {D}$ and $\bar{\text {D}}$ mesons interact with the hadrons that compose the thermal bath. We assume that the main contribution to the drag force and diffusion coefficients is due to their scattering with the most abundant hadronic species. For the microscopic calculation of transport coefficients we consider the set of pseudoscalar light mesons $\pi$, K, $\bar{\text {K}}$, $\eta$ and the baryons N, $\bar{\text {N}}$, $\Delta$, $\bar{\Delta }$.

A detailed presentation of the effective Lagrangian for heavy mesons and transport coefficients is described in Refs. [67–70]. Here we only review the basic aspects of the methodology. We split the discussion between the interaction of $\text {D}$ mesons with lighter mesons, and with baryons. The two sectors have in common that the effective Lagrangian follows from the principles of chiral and heavy-quark spin symmetry (HQSS), and the final scattering matrix elements satisfy exact unitarity constraints. Unitarity is assured by the implementation of a unitarization procedure to the perturbative scattering amplitudes obtained from the effective theory.

#### Interaction with light mesons

The effective Lagrangian describing $\text {D}$ mesons and the light pseudoscalar mesons is described in Refs. [67, 69] (and references therein). The $\text {D}$ meson is incorporated within a $J=0$ isotriplet $D=(D^0,D^+,D^+_s)$. In addition, the $J=1$ meson field $D^*_\mu =(D^{*0},D^{*+},D_{s}^{*+})_\mu$ is also introduced in accordance to HQSS. The set of $\text {SU}(3)_f$ (pseudo-)Goldstone bosons is introduced via the exponential representation $U=u^2=\exp \left( \frac{\sqrt{2} \text {i}\Phi }{f} \right)$, where the matrix

21 $$\begin{aligned} \Phi = \left( \begin{array}{ccc} \frac{1}{\sqrt{2}} \pi ^0 + \frac{1}{\sqrt{6}} \eta &{} \pi ^+ &{} K^+ \\ \pi ^- &{} - \frac{1}{\sqrt{2}} \pi ^0 + \frac{1}{\sqrt{6}} \eta &{} K^0 \\ K^- &{} {\bar{K}}^0 &{} - \frac{2}{\sqrt{6}} \eta \end{array} \right) \ , \end{aligned}$$

and *f* is the pion decay constant in the chiral limit. The leading-order (LO) Lagrangian is fixed by chiral symmetry and HQSS. It incorporates the standard LO chiral perturbation theory for the Goldstone bosons, and

22 $$\begin{aligned} \mathscr {L}_{{LO}}= & {} \langle \nabla ^\mu D \nabla _\mu D^\dag \rangle - m_{\text {D}}^2 \langle DD^{\dag } \rangle - \langle \nabla ^\mu D^{*\nu } \nabla _\mu D^{*\dag }_{\nu } \rangle \nonumber \\&+ m_{{\text {D}}}^2 \langle D^{*\mu } D_\mu ^{* \dag } \rangle + ig \langle D^{* \mu } u_\mu D^\dag - D u^\mu D_\mu ^{*\dag } \rangle \nonumber \\&+ \frac{g}{2m_{\text {D}}} \langle D^*_\mu u_\alpha \nabla _\beta D_\nu ^{*\dag } - \nabla _\beta D^*_\mu u_\alpha D_\nu ^{*\dag }\rangle \epsilon ^{\mu \nu \alpha \beta }\ , \end{aligned}$$

where $m_{\text {D}}$ is the tree-level heavy-meson mass, the bracket denotes trace in flavor space, and

23 $$\begin{aligned} u_\mu&= \text {i}\left( u^\dag \partial _\mu u - u \partial _\mu u^\dag \right) \, , \end{aligned}$$

24 $$\begin{aligned} \nabla _\mu&= \partial _\mu - \frac{1}{2} \left( u^\dag \partial _\mu u + u \partial _\mu u^\dag \right) \, , \end{aligned}$$

are the auxiliary axial vector field, and the covariant derivative, respectively. The coupling *g* connects heavy and light mesons and can be fixed such that the decay width of the process $\text {D}^* \rightarrow \text {D}+\pi$ is reproduced. The Lagrangian is further expanded up to next-to-leading order (NLO) in chiral counting. This order (not reproduced here) is needed to account for the light-meson masses and additional interactions between heavy and light sectors. The expression of the perturbative potential at NLO is

25 $$\begin{aligned} V^{\text {meson}}_{ij}= & {} \frac{C_{0,ij}}{2 f^2} (p_1 \cdot p_2 - p_1 \cdot p_4) + \frac{2C_{1,ij} h_1}{f^2} \nonumber \\&+ \frac{2C_{2,ij}}{f^2} h_3 (p_2 \cdot p_4) + \frac{2C_{3,ij}}{f^2} h_5 \nonumber \\&\times \left[ (p_1 \cdot p_2) (p_3 \cdot p_4) + (p_1 \cdot p_4) (p_2 \cdot p_3) \right] \ ,\nonumber \\ \end{aligned}$$

where *i*, *j* denote the incoming and outgoing scattering channels ($1,2 \rightarrow 3,4$), $C_{n,ij}$ are numerical coefficients depending on the isospin, spin, strangeness and charm quantum numbers, and $h_n$ are the low-energy coefficients, appearing at NLO and not fixed by symmetry arguments alone, but by matching physical observables to experimental data [67]. Equation (25) provides the NLO scattering amplitudes for meson–meson (elastic and inelastic) scattering. The interactions of $\bar{\text {D}}$ mesons are obtained by appropriate charge conjugations.

To increase the validity to moderate energies we impose exact unitarity on these amplitudes. This is achieved by the solution of the Bethe-Salpeter equation, or *T*-matrix approach similar to the one used for the partonic case. We use *V* as the kernel for the $T-$matrix equation, in a full coupled-channel basis. The integral equation is simplified within the “on-shell” approximation [67] and transformed into an algebraic equation $T=V+V\tilde{G}T$, which is readily solved by

26 $$\begin{aligned} T_{ij} = [1-V\tilde{G}]_{ik}^{-1} V_{kj} \ , \end{aligned}$$

where $\tilde{G}$ is the so-called loop function (integral over the internal momentum of the two-particle propagator).

In addition to the exact unitarity satisfied by *T*, the unitarization method produces a set of resonance and bound states in some of the scattering channels, appearing as poles in the complex-energy plane of *T*. The identification of these poles with experimental states, helps us to fix the unknown parameters of the effective approach (low-energy constants and the regularization parameters of $\tilde{G}$). In particular, we obtain the $D_0^*(2400)$ in the $(I,J^P)=(1/2,0^+)$ channel, and the bound state $D^*_{s0}(2317)$ in the $(I,J^P)=(0,0^+)$.

**Fig. 3:**
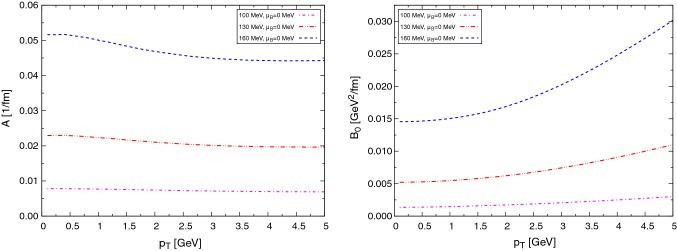
Drag (left) and diffusion (right) coefficients at different temperatures for $\text {D}$ mesons interacting with the pseudoscalar meson octet $\pi$, *K*, $\bar{K}$, $\eta$

In Fig. 3 we present the drag force (left panel) and diffusion coefficient (right panel) of $\text {D}$ mesons interacting with light mesons as functions of momentum for several temperatures at $\mu _{\text {B}}=0$. For large momentum – beyond the natural application of the effective Lagrangian – the interactions are taken assuming constant cross sections. Although the qualitative behavior of the transport coefficients is similar to the case for *c* quarks, notice that the numerical values are one order of magnitude smaller.

#### Interaction with baryons

The interaction of $\text {D}$ mesons with baryons follows a parallel methodology using an effective Lagrangian based on chiral and HQSS symmetries. In this case the formalism is taken from Refs. [70–74]. The Lagrangian is considered at LO in chiral expansion, and is further reduced to a Weinberg–Tomozawa interaction when the Goldstone bosons participate in the interaction. Then, the $\text {SU}(3)_f$ chiral symmetry is enlarged to $\text {SU}(6)$ symmetry (spin times flavor). From the degrees of freedom introduced in the effective description, we focus on those involved in the interaction of the $\text {D}$ meson with $N,{{\bar{N}}}, \Delta$ and ${\bar{\Delta }}$ baryons.

The tree-level meson–baryon scattering amplitudes have the structure

27 $$\begin{aligned} V_{ij}^{\text {baryon}}= & {} \frac{D_{ij}}{4\,f_i f_j} (2\sqrt{s}-M_i-M_j) \nonumber \\&\times \sqrt{\frac{M_i+E_i}{2M_i}}\sqrt{\frac{M_j+E_j}{2M_j}} \ , \end{aligned}$$

where $M_i$, $E_i$ and $f_i$ denote respectively the baryon mass, C.M. energy, and the meson decay constant participating in the *i* channel. The $D_{ij}$ are numerical coefficients depending on the quantum numbers of the scattering channel.

As in the meson sector, these amplitudes are used as kernels in a coupled-channel *T*-matrix approach. It is again solved in the “on-shell” approximation to obtain the solution given of Eq. (26), which satisfies exact unitarity. A large set of resonant and bound states are dynamically generated by the unitarization procedure. The most prominent ones being the $\Lambda _c (2595)$ in the $(I,J^P)=(0,1/2^-)$ channel and the $\Sigma _c (2550)$ in the $(I,J^P)=(1,3/2^-)$ channel.

Once the scattering amplitudes are fixed, the $\text {D}$-meson transport coefficients are computed – like in the partonic case – within the Fokker-Planck approximation. The drag force and the diffusion coefficients are calculated using the same equations as in (18, 20), but implementing quantum statistics instead. Pertinent isospin-spin degeneracy factors are used for each degree of freedom.

The dependence of the transport coefficients on the chemical potential has been addressed in Ref. [69]. To an excellent approximation the fugacity ($z=\text {e}^{\mu _B/T}$) factorizes out of the expression of the meson–baryon transport coefficients (and $z^{-1}$ factorizes out for the antibaryon case). In this respect, the transport coefficients of the $\text {D}$ meson can be constructed by a linear combination of the transport coefficients of mesons, baryon and antibaryon at $\mu _B=0$, with respective coefficients 1, *z*, $z^{-1}$ (for the $\bar{\text {D}}$ meson, baryon and antibaryon coefficients should be reversed).

**Fig. 4:**
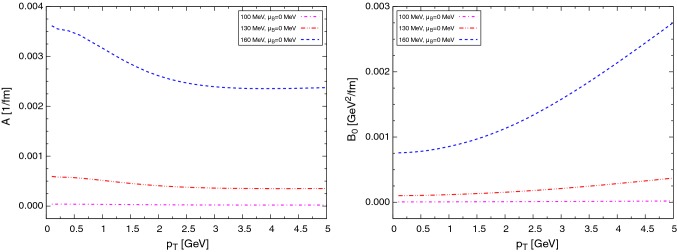
Drag (left) and diffusion (right) coefficients at different temperatures for $\text {D}$ mesons interacting with baryons *N* and $\Delta$

In Fig. 4 we show the transport coefficients for the $\text {D}$ mesons interacting with baryons (alternatively, $\bar{\text {D}}$ with antibaryons). Due to the Boltzmann suppression of baryons, the transport coefficients are considerably suppressed with respect to those for mesons.

**Fig. 5:**
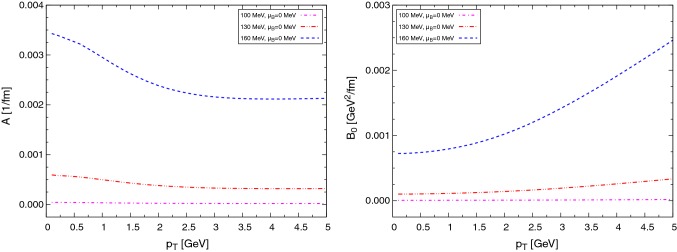
Drag (left) and diffusion (right) coefficients at different temperatures for $\bar{\text {D}}$ mesons interacting with baryons *N* and $\Delta$

In Fig. 5 we present a similar plot of the coefficients for the $\bar{\text {D}}$ mesons interacting with baryons (equivalently, $\text {D}$ mesons with antibaryons). Let us note that for the rather different cross sections as compared to the previous case, the transport coefficients are very similar. The reason is that the transport coefficients are not very sensitive to the details of the scattering amplitude (resonance peaks, channel openings...), but only to the thermal average of it, which is similar in both cases. However, we note that the $\text {D}$-meson–baryon interaction is stronger, with more resonances contributing to the total cross section. This is reflected in slightly larger coefficients.

**Fig. 6:**
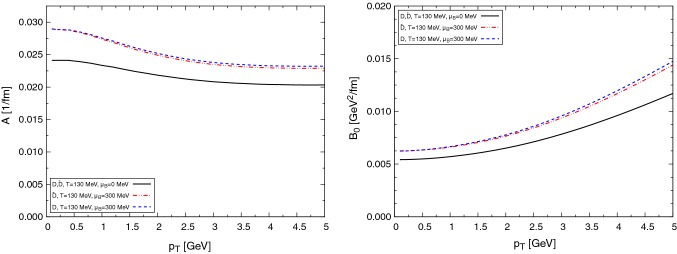
Drag (left) and diffusion (right) coefficients at different temperatures for $\text {D}$ and $\bar{\text {D}}$ mesons, taking into account a fugacity factor

**Fig. 7:**
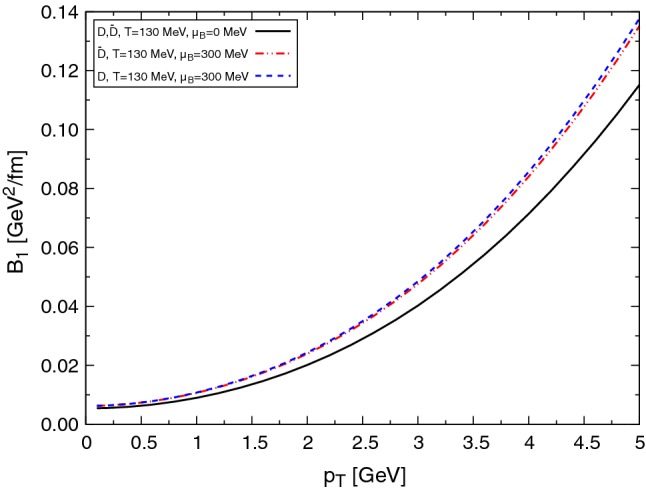
$B_1$ coefficients at different temperatures for $\text {D}$ and $\bar{\text {D}}$ mesons, taking into account a fugacity factor

Finally, in Figs. 6 and 7 we show the effect of the baryochemical potential. A sizable increase of the drag and diffusion coefficients is obtained for moderate values of the chemical potential, entirely due to the baryon and antibaryon contributions. For higher $\mu _{\text {B}}$ this important increase of the coefficients produces a large energy loss and momentum diffusion of $\text {D}$ mesons in dense matter.

## Implementation of the numerical simulations

Notice: except in case of explicit distinctions, in this section we will use the term *c*, *charm* quark and $\text {D}$ mesons both for particles and anti-particles. More precisely, we will consider $D^+$ and $D^-$ only, excluding all other open charm mesons.

**Fig. 8:**
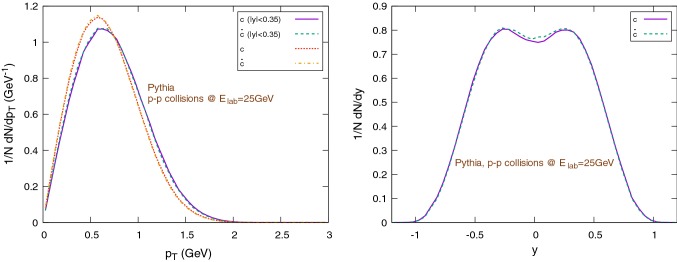
Spectra of initial charms and anti-charms as sampled with Pythia. The left figure shows the (normalized) $1/N \text {d}N/\text {d}p_{\text {T}}$ distribution (in the rapidity range $|y|<0.35$), the right figure shows the (normalized) $1/N \text {d}N/ \text {d}y$ distribution

**Fig. 9:**
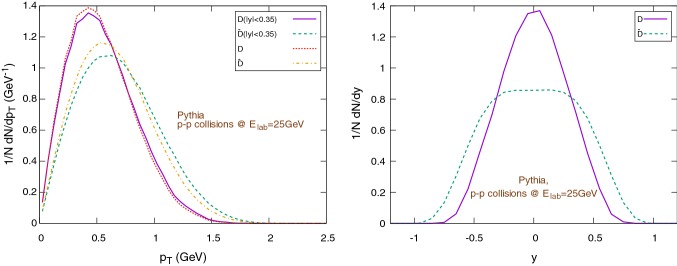
Spectra of $\text {D}/\bar{\text {D}}$-mesons in p–p collisions sampled with Pythia. The left figure shows the (normalized) $1/N \text {d}N/\text {d}p_{\text {T}}$ distribution (in the rapidity range $|y|<0.35$), the right figure shows the (normalized) $1/N \text {d}N/\text {d}y$ distribution

We use Pythia 8.2 [75, 76] to obtain a set of $10^6$ charm-anti-charm quark pairs by performing p+p collisions at $E_{\text {lab}}=25\,\text {GeV}$, enabling the *SoftQCD* mode.^2^ The initial charm and anti-charm distributions versus transverse momentum and rapidity are shown in Fig. 8. Pythia is also used to compute the $\text {D}/\bar{\text {D}}$ mesons momentum distribution in $p-p$ collisions, shown in Fig. 9 with respect to the transverse momentum (left) and rapidity (right). Here one observes (Fig. 9, right) that the different production channels $\text {pp} \rightarrow \text {D}\bar{\text {D}}+X$ and $\text {pp} \rightarrow \bar{\text {D}}\Lambda _c+X$ lead to different initial rapidity distributions for the charm and anti-charm channels.

After this preliminary step, we perform the Langevin propagation of the charm quarks in the background medium, first modeling it with the UrQMD hydrid model [27] and then with the UrQMD coarse-graining approach [28].

To obtain the space-time points of the production of the charm quarks, we perform an UrQMD run with elastic zero degree scatterings between the colliding nuclei (Monte Carlo Glauber initial conditions), saving the space-time coordinates of the points where collisions between the nucleons happened. In the subsequent full UrQMD runs, for each event we distribute over these collision points around 140,000 *c*-$\bar{c}$ pairs previously created with Pythia. The (anti-)charm quarks propagate along straight lines without interacting with any particle until the onset of the hydrodynamical phase, i.e. after the two nuclei have completely passed through each other at $t=(2R_{\text {nucl}})/(\sqrt{\gamma ^2_{\text {CM}}-1})\approxeq 3.5\,\text {fm}$. The timestep for the Langevin propagation is $\text {d}t_{\text {Langevin}}=0.01\, \text {d}t_{\text {hydro}}$ for each hydro timestep. We have checked that this accuracy is sufficient to obtain stable results. At each Langevin iteration step we use the values of the fluid temperature *T* and fluid velocity components $v_i$ to perform a bilinear interpolation of the transport coefficients (which depend on the momentum *p* and the temperature *T*). The finite baryon chemical potential is taken into account by multiplying the drag and diffusion coefficients of the charm quarks by a fugacity factor $\text {e}^{\mu _q/T}$ for $\bar{c}$ quarks $\text {e}^{-\mu _q/T}$ for *c* quarks ($\mu _q=\mu _B/3$). For the $\text {D}$ mesons we use $K_{\text {D}}(T,\mu _B,p)=K_{\text {mesons}}^{\text {D}}(T,p)+\text {e}^{\mu _B/T}K^{\text {D}}_{\text {baryons}}(T,p)+\text {e}^{-\mu _B/T}K^{\text {D}}_{\text {antibar}}(T,p)$ and $K_{\bar{\text {D}}}(T,\mu _B,p)=K^{\text {D}}_{\text {mesons}}(T,p)+\text {e}^{-\mu _B/T}K^{\text {D}}_{\text {baryons}}(T,p)+\text {e}^{\mu _B/T}K^{\text {D}}_{\text {antibar}}(T,p)$, where the *K* is any of the transport coefficients *A*, $B_\perp$, $B_\parallel$ and $K^{\text {D}}_{\text {mesons}}$, $K^{\text {D}}_{\text {baryons}}$, $K^{\text {D}}_{\text {antib}}$ are the contributions coming from the interactions of $\text {D}$ mesons with other mesons, baryons and anti-baryons, respectively. In our model, we assume that the medium affects the propagation of the heavy quarks, but the medium itself is not affected by the heavy quarks that we inject. There is also no interaction between the injected charm quarks. This approximation allows us to use a large number of charm quarks per event, thus reducing considerably the number of events needed to reach a sufficient statistics.

We assume to have instantaneous hadronization and decoupling processes which happen at the same temperature $T_c$, that means that the $c(\bar{c})$ quarks immediately become $\text {D}(\bar{\text {D}})$-mesons as soon as they are found to be in a fluid cell with a temperature $T<T_c$ and, on the contrary, $\text {D}(\bar{\text {D}})$-mesons become $c(\bar{c})$ quarks if they are in a cell with $T>T_c$.

We consider hadronization either through coalescence or Peterson fragmentation. We assume a constituent quark rest mass for up and down quarks of $m_{u,d}=369\,\text {MeV}$, a charm quark mass $m_c=1.5\,\text {GeV}$ and a $\text {D}$-meson mass $m_{\text {D}}=1.869\,\text {GeV}$ (we neglect the $5\,\text {MeV}$ mass difference between $\text {D}^+/\text {D}^-$ and $\text {D}^0/\bar{\text {D}}^0$). The velocity components $v_x, v_y, v_z$ of the light quarks are taken as equal to the fluid velocity, i.e. thermal smearing is omitted. The probability of hadronization by coalescence, $P_{\text {coa}}$ [77], in terms of the four momentum components $p^{\mu }$ of the light quarks is given by:

28 $$\begin{aligned} P_{\text {coa}}=\exp \left\{ \left[ (\Delta p^0)^2-\sum \limits _{i=1}^3(\Delta p^i)^2-(\Delta _m)^2 \right] \sigma ^2\right\} . \end{aligned}$$

Here the $\Delta p$ are the differences between the four-momentum components of the heavy and the light quark, $\Delta _m=m_{{c}} - m_{{u,d}}$, $\sigma =\sqrt{\frac{8}{3(\hbar {c})^{2}} r^{2}_{\text {D}_{(\mathrm{rms})}}}$ and $r^{2}_{\text {D}_{(\mathrm{rms})}}$ is the mean squared radius of the $\text {D}$-meson.

In case of coalescence, the four-momentum of the newly formed $\text {D}(\bar{\text {D}})$-meson is given by the sum of the four-momenta of the constituent quarks, while, in case of Peterson fragmentation, the $\text {D}(\bar{\text {D}})$-meson obtains a fraction of momentum of the charm quark according to the distribution [78]:

29 $$\begin{aligned} D(z)=\frac{H}{z[1-(1/z)-\epsilon _p/(1-z)]^2}. \end{aligned}$$

Here *H* is a normalization constant, *z* the momentum fraction obtained in the fragmentation and $\epsilon _p$ a parameter. Peterson fragmentation is the only process allowed for heavy quarks hadronizing in the void, a condition that may occur in the coarse graining approach. On average, roughly $80\%$ of the times the hadronization channel is Peterson fragmentation, more than it is commonly expected at low collision energies, especially if we consider that in Eq. (28) we removed the dependence on the spatial distance between the heavy and the light quark in the probability distribution, which is present, for example, in the original Ref. [77] or in Ref. [17]. At the moment, we do not have a clear explanation for this issue.

In the case of the charm quarks originating from $\text {D}$ mesons entering into cells with $T>T_c$, we maintain the four-velocity. We evolve the UrQMD hydro simulations until the energy density over the grid is below $0.3\varepsilon _0$ ($\varepsilon _0=146.5\,\text {MeV}/\text {fm}^3$), then, using the phase-space data (position and velocities) of the charm quarks at the beginning of the hydro phase, we repeat each series using the coarse-graining approach.

We maintain the same time step for the Langevin propagation process that we use in hydro, i.e. $8\cdot 10^{-4}\,\text {fm}$, so, since the time resolution of the coarse graining data is $0.2\,\text {fm}$ for reactions with impact parameter $b=3\,\text {fm}$ and $0.4\,\text {fm}$ for reactions with impact parameter $b=3\,\text {fm}$, we perform 250 and 500 iterations per coarse-graining time step, respectively. As before for the hydro case, we check that the choice of the time step has no effect on the final results. We start the simulations in the coarse-graining approach from $3.6\,\text {fm}$, propagating again the charm quarks along straight lines from the hydro starting time until this time. The method is the same as in the hydrid approach. However, in addition we can now follow the bulk evolution of the system until $t=75\,\text {fm}$. To avoid spurious effects in the coarse-graining simulations due to a few cells with low statistics and therefore unrealistic momentum transfers, we limit the fugacity factors to lie in the range [0.01–100], after a comparison with the hydro case.

The $\text {D}$ mesons decay weakly into non charmed hadrons before reaching the detectors, however they are relatively long-lived, with proper mean decay lengths of order $100\,\upmu \text {m}$ [79], therefore their decay products are not affected by hadronic rescattering and the decay vertices can be accurately reconstructed. This is the reason why we did not consider important to simulate also their decay into directly observed hadrons. However, probably we will reconsider open heavy flavor meson decays in future studies, when including also excited states [80], or when working at higher collision energies and interested in distinguishing the prompt $\text {D}$ mesons signal from the feed-down of heavier particles. For each combination of the parameters, we run 2000 events.

## Results

We simulate Au+Au reactions at $E_{\text {lab}}=25\,A\text {GeV}$ at fixed impact parameters $b=3\,\text {fm}$ and $b=7\,\text {fm}$. The simulations are performed both for the hybrid set-up and the coarse-graining approach. The elliptic flow at mid-rapidity is calculated in the reaction plane as:

30 $$\begin{aligned} v_2(p_{\text {T}})=\left\langle \dfrac{p_x^2-p_y^2}{p_x^2+p_y^2}\right\rangle ,\quad |y|<0.35, \end{aligned}$$

where *E*, $p_x$, $p_y$ and $p_z$ are the four-momentum components, $p_{\text {T}}=\sqrt{p_x^2+p_y^2}$ is the transverse momentum, $y=1/2\ln [(E+p_z)/(E-p_z)]$ is the rapidity and the averages are made over all charm particles produced in all the events of a series at a given impact parameter.

**Fig. 10:**
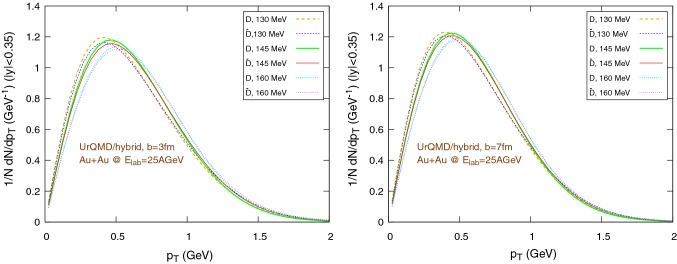
Normalized $1/N \text {d}N/\text {d}p_{\text {T}}$ distribution of the final $\text {D}/\bar{\text {D}}$ mesons, in the rapidity range $|y|<0.35$, for Au+Au collisions at $E_{\text {lab}}=25\,\text {AGeV}$, using the UrQMD/hybrid model. The hadronization parameters are $\epsilon _p=0.05$ and $\left\langle {r_{\text {D}_{\text {rms}}}} \right\rangle =0.6\,\text {fm}$. Left: $b=3\,\text {fm}$, right: $b=7\,\text {fm}$

**Fig. 11:**
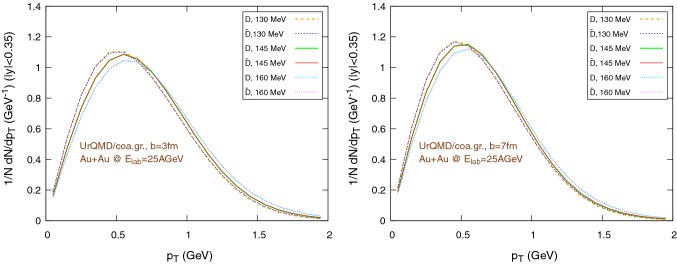
Normalized $1/N \text {d}N/\text {d}p_{\text {T}}$ distribution of the final $\text {D}/\bar{\text {D}}$ mesons, in the rapidity range $|y|<0.35$, for Au+Au collisions at $E_{\text {lab}}=25\,\text {AGeV}$, using the UrQMD/coarse-graining approach. The hadronization parameters are $\epsilon _p=0.05$ and $\left\langle {r_{\text {D}_{\text {rms}}}} \right\rangle =0.6\,\text {fm}$. Left: $b=3\,\text {fm}$, right: $b=7\,\text {fm}$

**Fig. 12:**
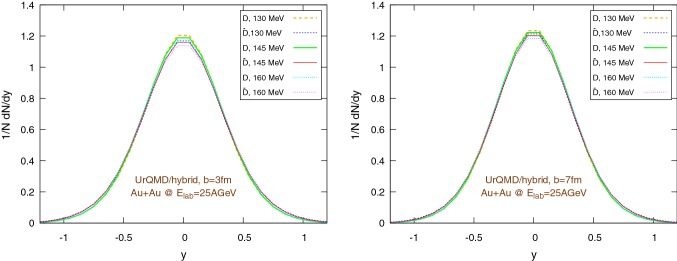
Final $1/N \text {d}N/\text {d}y$ distribution using the UrQMD/hybrid model for Au+Au collisions at 25 GeV per nucleon in the lab frame, assuming different hadronization temperatures, with fixed parameters: $\epsilon _p=0.05$ and $\left\langle {r_{\text {D}_{\text {rms}}}} \right\rangle =0.6\,\text {fm}$. Left: $b=3\,\text {fm}$, right: $b=7\,\text {fm}$

**Fig. 13:**
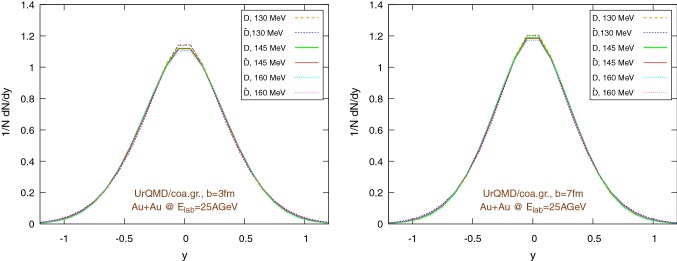
Final $1/N \text {d}N/\text {d}y$ distribution using the UrQMD/coarse-graining approach for Au+Au collisions at 25 GeV per nucleon in the lab frame, assuming different hadronization temperatures, with fixed parameters: $\epsilon _p=0.05$ and $\left\langle {r_{\text {D}_{\text {rms}}}} \right\rangle =0.6\,\text {fm}$. Left: $b=3\,\text {fm}$, right: $b=7\,\text {fm}$

**Fig. 14:**
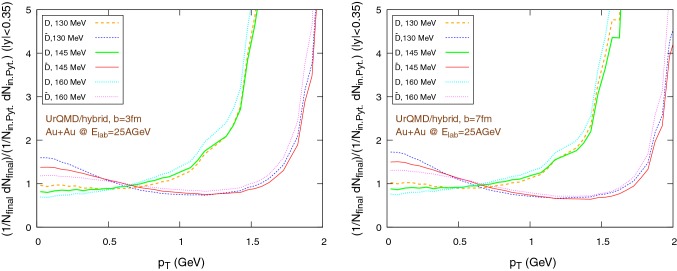
$\tilde{R}_{AA}$, i.e. the ratio of the individually normalized distributions $1/N_{\text {final}} \text {d}N_{\text {final}}/\text {d}p_{\text {T}}$ in Au+Au collisions and $1/N_{\text {in. Pyt.}} \text {d}N_{\text {in. Pyt.}}/\text {d}p_{\text {T}}$ in pp collisions (simulated with Pythia), in the rapidity range $|y|<0.35$, for Au+Au collisions at $E_{\text {lab}}=25\,\text {AGeV}$, using the UrQMD/hybrid model. The hadronization parameters are $\epsilon _p=0.05$ and $\left\langle {r_{\text {D}_{\text {rms}}}} \right\rangle =0.6\,\text {fm}$. Left: $b=3\,\text {fm}$, right: $b=7\,\text {fm}$

**Fig. 15:**
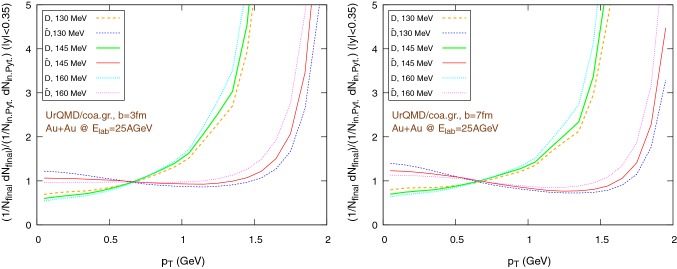
$\tilde{R}_{AA}$, i.e. the ratio of the individually normalized distributions $1/N_{\text {final}} \text {d}N_{\text {final}}/\text {d}p_{\text {T}}$ in Au+Au collisions and $1/N_{\text {in. Pyt.}} \text {d}N_{\text {in. Pyt.}}/\text {d}p_{\text {T}}$ in pp collisions (simulated with Pythia), in the rapidity range $|y|<0.35$, for Au+Au collisions at $E_{\text {lab}}=25\,\text {AGeV}$, using the UrQMD/coarse-graining approach. The hadronization parameters are $\epsilon _p=0.05$ and $\left\langle {r_{\text {D}_{\text {rms}}}} \right\rangle =0.6\,\text {fm}$. Left: $b=3\,\text {fm}$, right: $b=7\,\text {fm}$

**Fig. 16:**
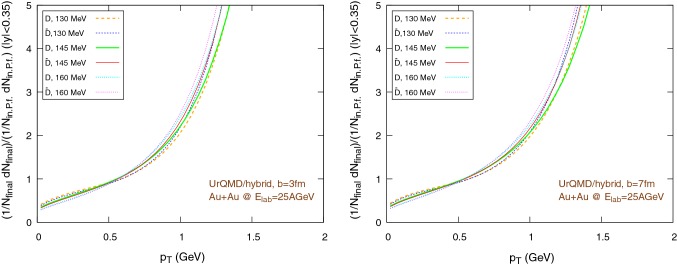
$\tilde{R}_{AA}$, i.e. the ratio of the individually normalized distributions $1/N_{\text {final}} \text {d}N_{\text {final}}/\text {d}p_{\text {T}}$ in Au+Au collisions and $1/N_{\text {in. Pyt.}} \text {d}N_{\text {in. P. f.}}/\text {d}p_{\text {T}}$ in pp collisions (Pythia + Peterson fragmentation), in the rapidity range $|y|<0.35$, for Au+Au collisions at $E_{\text {lab}}=25\,\text {AGeV}$, using the UrQMD/hybrid model. The hadronization parameters are $\epsilon _p=0.05$ and $\left\langle {r_{\text {D}_{\text {rms}}}} \right\rangle =0.6\,\text {fm}$. Left: $b=3\,\text {fm}$, right: $b=7\,\text {fm}$

**Fig. 17:**
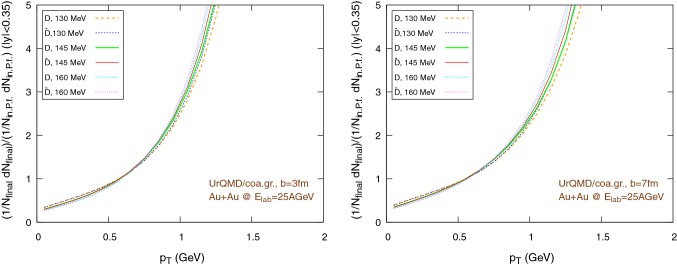
$\tilde{R}_{AA}$, i.e. the ratio of the individually normalized distributions $1/N_{\text {final}} \text {d}N_{\text {final}}/\text {d}p_{\text {T}}$ in Au+Au collisions and $1/N_{\text {in. Pyt.}} \text {d}N_{\text {in. P. f.}}/\text {d}p_{\text {T}}$ in pp collisions (Pythia + Peterson fragmentation), in the rapidity range $|y|<0.35$, for Au+Au collisions at $E_{\text {lab}}=25\,\text {AGeV}$, using the UrQMD/coarse-graining approach. The hadronization parameters are $\epsilon _p=0.05$ and $\left\langle {r_{\text {D}_{\text {rms}}}} \right\rangle =0.6\,\text {fm}$. Left: $b=3\,\text {fm}$, right: $b=7\,\text {fm}$

**Fig. 18:**
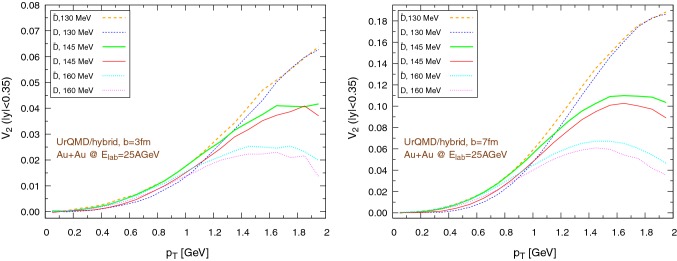
Elliptic flow of $\text {D}/\bar{\text {D}}$ mesons ($|y|<0.35$) within the UrQMD/hybrid approach in Au+Au collisions at $E_{\text {lab}}=25$ AGeV. We show different hadronization temperatures, with fixed parameters: $\epsilon _p=0.05$ and $\left\langle {r_{\text {D}_{\text {rms}}}} \right\rangle =0.6\,\text {fm}$. Left: $b=3\,\text {fm}$, right: $b=7\,\text {fm}$. (Note the different scales on the ordinate.)

**Fig. 19:**
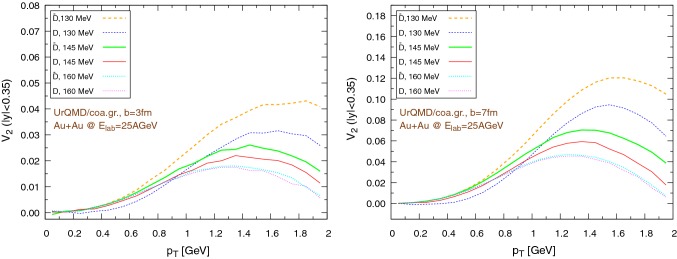
Elliptic flow of $\text {D}/\bar{\text {D}}$ mesons ($|y|<0.35$) within the UrQMD/coarse-graining approach in Au+Au collisions at $E_{\text {lab}}=25$ AGeV. We show different hadronization temperatures, with fixed parameters: $\epsilon _p=0.05$ and $\left\langle {r_{\text {D}_{\text {rms}}}} \right\rangle =0.6\,\text {fm}$. Left: $b=3\,\text {fm}$, right: $b=7\,\text {fm}$

**Fig. 20:**
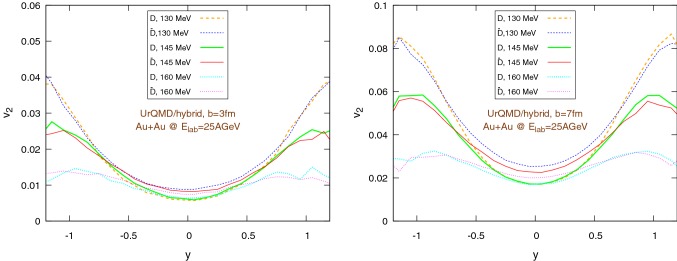
Elliptic flow of $\text {D}/\bar{\text {D}}$ mesons with respect to rapidity within the UrQMD/hybrid model in Au+Au collisions at $E_{\text {lab}}=25$ AGeV. We show different hadronization temperatures, with fixed parameters: $\epsilon _p=0.05$ and $\left\langle {r_{\text {D}_{\text {rms}}}} \right\rangle =0.6\,\text {fm}$. Left: $b=3\,\text {fm}$, right: $b=7\,\text {fm}$

**Fig. 21:**
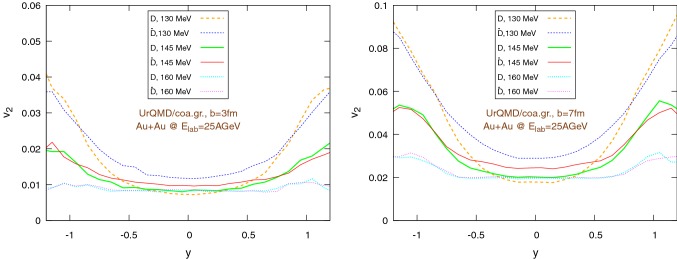
Elliptic flow of $\text {D}/\bar{\text {D}}$ mesons with respect to rapidity within the UrQMD/coarse-graining approach in Au+Au collisions at $E_{\text {lab}}=25$ AGeV. We show different hadronization temperatures, with fixed parameters: $\epsilon _p=0.05$ and $\left\langle {r_{\text {D}_{\text {rms}}}} \right\rangle =0.6\,\text {fm}$. Left: $b=3\,\text {fm}$, right: $b=7\,\text {fm}$

### Dependence on the hadronization temperature

To explore the sensitivity of the $\text {D}/\bar{\text {D}}$ elliptic flow and momentum distribution on the lifetime of the partonic phase, we evaluate the effect of three different hadronization temperatures: $160\,\text {MeV}$, $145\,\text {MeV}$ and $130\,\text {MeV}$. In all cases we perform the Langevin propagation until the local temperature of the computational cell is above $60\,\text {MeV}$. In Eq. (28), which gives the probability to hadronize by coalescence, we set $\left\langle {r_{\text {D}_{\text {rms}}}} \right\rangle =0.6\,\text {fm}$, while in the fragmentation function (Eq. 29) we set $\epsilon _p=0.05$.

Figures 10 and 11 show the transverse momentum distribution for the final $\text {D}/\bar{\text {D}}$ mesons in the UrQMD/hybrid model and in the UrQMD/coarse-graining approach, respectively, while Figs. 12 and 13 show their rapidity distributions.

Figures 14 and 15 show $\tilde{R}_{AA}$, i.e. $\tilde{R}_{AA}=\dfrac{1/N_{AA}\text {d}N/\text {d}p_{\text {T}} |_{AA}}{1/N_{\text {pp}}\text {d}N/\text {d}p_{\text {T}} |_{\text {pp}}}$, where the distribution in $\text {pp}$ is taken from (Fig. 9, left). In particular, Fig. 14 refers to the UrQMD/hybrid model, while Fig. 15 refers to the coarse graining approach. The left and right sides of the figures refer to reactions at fixed impact parameter $b=3\,\text {fm}$ and $b=7\,\text {fm}$, respectively. A general trend observed in both scenarios and for both impact parameters is the strong increase of $\tilde{R}_{AA}$ with increasing transverse momentum. This effect is due to energy conservation, which limits the maximum $p_{\text {T}}$ available in pp reactions to $p^{\text {max}}_T=(\sqrt{s_{\text {pp}}}-2m_p)/2\simeq 2.5\,\text {GeV}$. Therefore, we expect and observe this in the $R_{AA}$ as a strong increase.

To explore more in depth the uncertainties of the initial state, Figs. 16 and 17 show the same $\tilde{R}_{AA}$ distributions as before, however now with a different pp baseline. Instead of $\text {D}$-mesons from Pythia, we extract the charm quarks from Pythia in pp and hadronize them according to the Peterson fragmentation. As in the previous case, we observe a good consistency between the results coming from the UrQMD/hydro and the UrQMD/coarse-graining models. However, although essential features like the rise of $\tilde{R}_{AA}$ at “high” $p_T$ do not change when switching between the pp baselines, from a quantitative perspective there are noticeable differences. In particular, in Figs. 16 and 17 we miss the strong distinction between the $\tilde{R}_{AA}$ of particles and anti-particles visible in Figs. 14 and 15, due to the internal Pythia non-perturbative machinery and the inclusion of additional hadronization channels, already mentioned at the beginning of Sect. 5, which introduces a sharp difference in the spectra of *D* and $\bar{D}$ mesons, clearly shown in Fig. 9. On the other hand, it is well known that Pythia focuses on high-energy collisions and results at low energies obtained with non-tuned default program parameters should be taken with care. Anyway, the differences in the $\tilde{R}_{AA}$ depending on the chosen pp baseline suggest that Peterson fragmentation might tend to overlook important details of the hadronization process and they call for the development and/or the adoption of more sophisticated models. Regarding the normalized momentum distribution with respect to the rapidity, again we observe a good agreement between the UrQMD/hybrid model (Fig. 12) and the UrQMD/coarse-graining approach (Fig. 13). In both cases, for more central collisions we can observe a slightly more evident distinction between particles and anti-particles, in particular for lower hadronization temperature, associated with a small broadening of the distributions. These small effects are consistent with the expected larger interaction with the medium for $b=3\,\text {fm}$.

The results for the elliptic flow with respect to the transverse momentum are shown in Fig. 18, in the case of the UrQMD/hybrid model, and in Fig. 19, in the case of the UrQMD/coarse-graining approach. In all cases we observe that the elliptic flow of $\bar{\text {D}}$ is larger than the elliptic flow of $\text {D}$. As expected this is because of the fugacity factor which, in the partonic phase, enhances the transport coefficients for $\bar{\text {D}}$ and suppresses the transport coefficients for $\text {D}$. We also observe that the elliptic flow is higher for lower hadronization temperatures. With a larger time spent in the partonic phase, the larger magnitude of the transport coefficients in this phase compared to the hadronic phase leads to a stronger elliptic flow. By comparing $b=3\,\text {fm}$ and the $b=7\,\text {fm}$ collisions in Figs. 18 and 19 we notice that the $v_2$ for collisions having an impact parameter $b=7\,\text {fm}$ is larger than the $v_2$ for collisions with $b=3\,\text {fm}$. This behavior is consistent with the more anisotropic initial energy density spatial distribution in more peripheral collisions. By comparing Fig. 18 with Fig. 19, we observe that the $v_2$ in the case of the UrQMD/hybrid approach is larger than the $v_2$ in the case of the UrQMD/coarse-graining approach, showing the effects of the different viscosities in the two different modelings of the medium. In the UrQMD/coarse-graining approach the enhancement of the elliptic flow when switching from $b=3\,\text {fm}$ to $b=7\,\text {fm}$ is weaker than in the UrQMD/hybrid approach. This also indicates that partial thermalization might play a role.

Figures 20 and 21 show the dependence of the elliptic flow with respect to the rapidity. We observe that the $\bar{\text {D}}$ mesons have a significantly larger elliptic flow than the $\text {D}$ mesons only in the central rapidity region and for lower hadronization temperatures, in particular for peripheral collisions. Moreover, as a general trend, $v_2$ exhibits a minimum for $y=0$, nevertheless in the UrQMD/coarse-graining case the growth of $v_2$ moving away from the central rapidity region becomes important only for $|y|\gtrapprox 0.5$.

**Fig. 22:**
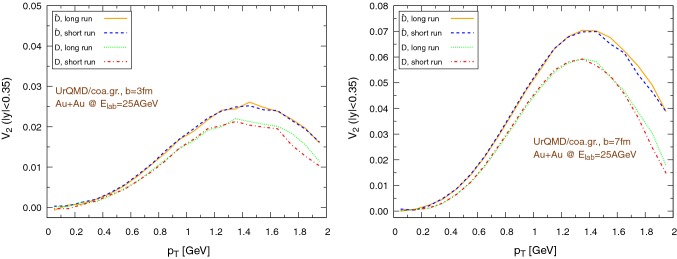
Au+Au collisions at $E_{\text {lab}}=25\,\text {AGeV}$ in the UrQMD/hybrid model. The hadronization parameters are $\epsilon _p=0.05$ and $\left\langle {r_{\text {D}_{\text {rms}}}} \right\rangle =0.6\,\text {fm}$. Left: $b=3\,\text {fm}$, right: $b=7\,\text {fm}$. Comparison between the elliptic flow of $\text {D}$ mesons ($|y|<0.35$) within the coarse-graining approach at two different final times: $75\,\text {fm}$ (*long run*) and $22\,\text {fm}$ (*short run*, $b=3\,\text {fm}$ ) or $19\,\text {fm}$ (*short run*, $b=7\,\text {fm}$)

**Fig. 23:**
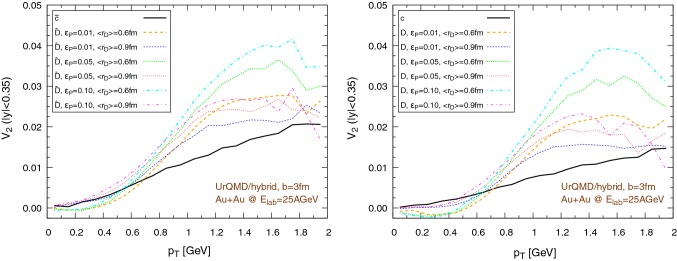
Au+Au collisions at $E_{\text {lab}}=25\,\text {AGeV}$, $b=3\,\text {fm}$ in the UrQMD/hybrid model. Elliptic flow of charm quarks and D-mesons ($|y|<0.35$). We explore the effect of different choices of the hadronization parameters, by performing a single hadronization process, without further hadronic propagation in the medium. Left: $\bar{c}$ quarks and $\bar{\text {D}}$ mesons, right: *c* quarks and $\text {D}$ mesons

**Fig. 24:**
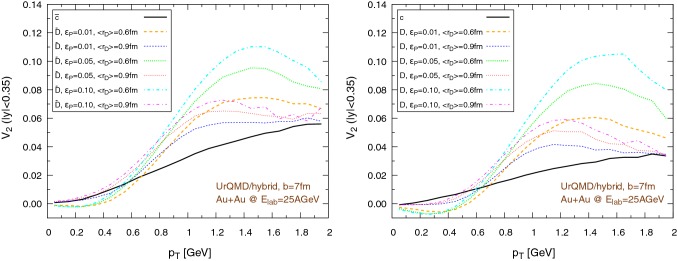
Au+Au collisions at $E_{\text {lab}}=25\,\text {AGeV}$, $b=7\,\text {fm}$ in the UrQMD/hybrid model. Elliptic flow of charm quarks and D-mesons ($|y|<0.35$). We explore the effect of different choices of the hadronization parameters, by performing a single hadronization process, without further hadronic propagation in the medium. Left: $\bar{c}$ quarks and $\bar{\text {D}}$ mesons, right: *c* quarks and $\text {D}$ mesons

**Fig. 25:**
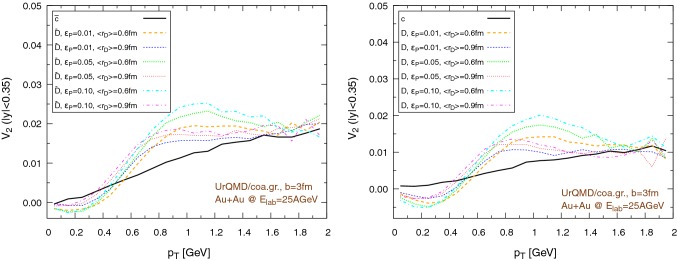
Au+Au collisions at $E_{\text {lab}}=25\,\text {AGeV}$, $b=3\,\text {fm}$ in the UrQMD/coarse-graining approach. Elliptic flow of charm quarks and D-mesons ($|y|<0.35$). We explore the effect of different choices of the hadronization parameters, by performing a single hadronization process, without further hadronic propagation in the medium. Left: $\bar{c}$ quarks and $\bar{\text {D}}$ mesons, right: *c* quarks and $\text {D}$ mesons

**Fig. 26:**
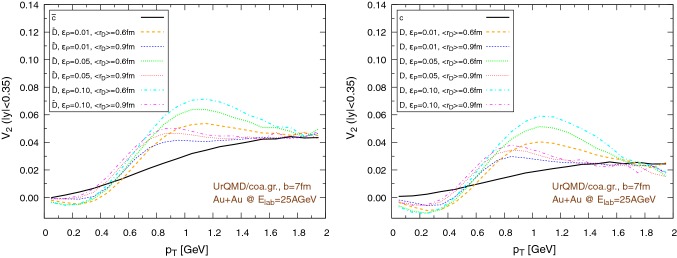
Au+Au collisions at $E_{\text {lab}}=25\,\text {AGeV}$, $b=7\,\text {fm}$ in the UrQMD/coarse-graining approach. Elliptic flow of charm quarks and D-mesons ($|y|<0.35$). We explore the effect of different choices of the hadronization parameters, by performing a single hadronization process, without further hadronic propagation in the medium. Left: $\bar{c}$ quarks and $\bar{\text {D}}$ mesons, right: *c* quarks and $\text {D}$ mesons

### The influence of the late hadronic phase

We recall that the final times in hybrid and coarse-graining approach are different: the condition to stop hydrodynamics (at maximum energy density of $0.3\varepsilon _0\approx 44\,\text {MeV}/\text {fm}^3$) is reached at $\approx 22\,\text {fm}$ for $b=3\,\text {fm}$ collisions and at $\approx 19\,\text {fm}$ for $b=7\,\text {fm}$ collisions, while the coarse-graining approach ends at $75\,\text {fm}$. It is important to stress that, since the hydro stopping temperature corresponding to $44\,\text {MeV}/\text {fm}^3$ is lower than $T_c$, the UrQMD/hybrid model always includes a hadronic phase, yet this is considerably shorter than in the UrQMD/coarse-graining approach. To evaluate the impact of this prolongated hadronic phase in the latter case, we repeat the $T_c=145\,\text {MeV}$ coarse-graining simulations at $E_{lab}=25\,\text {AGeV}$, with hadronization parameters $\epsilon _p=0.05$ and $\left\langle {r_{\text {D}_{\text {rms}}}} \right\rangle =0.6\,\text {fm}$, stopping them at the time of the average hydro ending time, i.e. $22\,\text {fm}$ for $b=3\,\text {fm}$ collisions and $19\,\text {fm}$ for $b=7\,\text {fm}$ collisions. We evaluate the elliptic flow of $\text {D}$ and $\bar{\text {D}}$ mesons at mid-rapidity, plotted in Fig. 22 both for $b=3\,\text {fm}$ (left) and for $b=7\,\text {fm}$ (right). In Fig. 22 the *long run* labels refer to simulations until $t=75\,\text {fm}$, while the *short* label refer to simulations terminated at $22\,\text {fm}$ (left) or $19\,\text {fm}$ (right). We can notice how the elliptic flow remains basically the same, in both centrality classes and both for $\text {D}$ and $\bar{\text {D}}$ mesons, except for small statistical fluctuations for $p_\text {T}\gtrsim 1.3\,\text {GeV}$. This means that the late hadronic phase does not alter the $\text {D}/\bar{\text {D}}$ distributions. This outcome confirms the expectations, because the transport coefficients for $\text {D}$ mesons are very small at low temperature, which in turn means that the $\text {D}$ mesons approach free streaming.

### The impact of the hadronization procedure

To assess the contribution of the partonic phase and the impact of the hadronization procedure on the flow, we perform the propagation of charm quarks until they reach for the first time a cell with temperature $T=T_c=145\,\text {MeV}$, then, without any further interaction with the medium, we hadronize the charm quarks. We further explore the effects of different values of the mean radius of the $\text {D}$ mesons $\left\langle {r_{\text {D}_{\text {rms}}}} \right\rangle$ ($0.6\,\text {fm}$ and $0.9\,\text {fm}$) and the Peterson fragmentation parameter $\epsilon _p$ (0.01, 0.05, 0.1). We recall that the assumptions on the size of the $\text {D}$ mesons play an important role in determining the probability of hadronization by coalescence or fragmentation, so different choices of $\left\langle {r_{\text {D}_{\text {rms}}}} \right\rangle$ correspond to different contributions of these two hadronization methods to $\text {D}$ meson formation. The results, for Au+Au collisions at $E_\mathrm{lab}=25\,\text {AGeV}$, are shown in Figs. 23, 24, 25 and 26. More precisely, the results of the UrQMD/hybrid model are shown in Fig. 23 for collisions at impact parameter $b=3\,\text {fm}$ and in Fig. 24 for collisions at $b=7\,\text {fm}$. The results of the UrQMD/coarse-graining approach are shown in Fig. 25 for collisions at $b=3\,\text {fm}$ and in Fig. 26 for collisions at $b=7\,\text {fm}$. All figures show the elliptic flow of quarks (solid black lines) at the moment of hadronization and of $\text {D}$ mesons (colored dashed lines) immediately after their formation. The left figures refer to $\bar{c}$ quarks and $\bar{\text {D}}$ mesons, the right figures to *c* quarks and *D* mesons. As an expected general trend, the $v_2$ of anti-particles is greater than the $v_2$ of particles. We observe that most of the flow is built during the partonic phase, a behavior consistent with the larger values of the transport coefficients at high temperatures. In addition, the difference in the magnitude of the flow between the hydro and the coarse-graining approach is clearly visible even at this stage. This implies that the use of the UrQMD/hybrid model down to temperatures at the limits of QGP existence is not the main responsible of the larger elliptic flow obtained in this model compared to the UrQMD/coarse-graining approach. Therefore, the suspect of an overestimation of $v_2$ due to a misuse of hydrodynamics is strongly reduced. Finally, in all cases, the elliptic flow grows with increasing values of $\epsilon _p$ and it is larger for smaller values of the $\text {D}$ meson radius.

It is clear that the details of the hadronization process have a very large impact on the final results, therefore special attention must be paid to a proper treatment of this step in future works. To begin, the probability distribution in Eq. (28) seems to overestimate of the probability to hadronization by fragmentation with the current choice of the $\text {D}$ meson radius, which might lead to wrong results, in particular when taking into account the formation of resonances with larger radii, especially if the dependence on the mutual spatial distance between the light and the heavy quark was also included [17, 77]. Apart for an extensive and deep re-checking of the whole procedure and its implementation in the code to better understand the origin of the apparently small percentage of hadronization by coalescence, we might replace Eq. (28) with a tabulated probability distribution obtained from full transport model simulations. Another possibility might be the adoption of a probability distribution which depends on the module of the relative velocity $|v_r|$ between the heavy quark and the fluid cell, i.e. something like $f(|v_r|)=\exp (-|v_r|/\alpha )$, with $\alpha$ determined by a fit with the elliptic flow measured in experiments at comparable collision energies. In addition, to be consistent with the assumptions made for the computation of the drag and diffusion coefficients in the partonic phase, we should go beyond the naive assumption of instantaneous hadronization and decoupling processes by introducing some probability function depending not only on temperature and chemical potential, but also explicitly on time. Indeed, the survival of $\text {D}$ mesons in the Quark Gluon Plasma might lead to a reduction of the predicted elliptic flow. Then, we should consider the probable formation of intermediate excited states and we should try to constrain the estimates of the $\text {D}$ meson radius, possibly making it also temperature dependent [81]. Moreover, we should try to improve the fragmentation process either by constraining the Peterson fragmentation parameter [82] or by adopting other fragmentation models [83], which in some cases have shown a better capability to reproduce the features of experimental data [84]. Further refinements might include medium modified [85] and unfavored [86] fragmentation functions. However, unfortunately, at the moment we miss well determined values of fragmentation functions for $\text {D}$ mesons in the low collision energy regime based on robust experimental data. Regarding the coalescence mechanism, the method itself is quite standard and the flow contribution to the final momentum of the open heavy meson is derived from the reliable UrQMD model, therefore the uncertainties are somehow reduced compared to the fragmentation mechanism. Nevertheless, although in this study we did not explore the consequences of different assumptions, the results depend on the estimates of the masses of the constituent quarks, which indirectly enter also in Eq. (28), therefore, even in this case, different educated choices of the parameters might alter the current predictions.

## Discussions and conclusion

In this paper we have presented results on $\text {D}$ and $\bar{\text {D}}$ meson spectra and elliptic flow for Au+Au reactions at $E_{\text {lab}}=25\,\text {AGeV}$. These calculations are relevant for the upcoming FAIR and NICA facilities and for the RHIC BES program. We have used Pythia [75, 76] to obtain a sample of correlated charm and anti-charm quarks, then we let the charm quarks propagate in the medium produced by heavy ion collisions, both in the partonic and in the hadronic phase, adopting a Langevin approach. In particular, we have studied Au+Au collisions at two different centralities, $b=3\,\text {fm}$ and $b=7\,\text {fm}$. The background medium is modeled either with the UrQMD hybrid model or with the UrQMD coarse graining approach. The effect of the finite baryon chemical potential is taken into account in the evaluation of the transport coefficients. The effect of different hadronization parameters is explored. We have shown that even at low collision energies the interaction with the medium produces a sizeable final $\text {D}$ meson elliptic flow, which is larger for more peripheral collisions. A lower decoupling temperature leads to an increase of the elliptic flow. This implies that the interaction with the medium is stronger during the partonic than during the hadronic phase. This hypothesis is also confirmed by the magnitude of the elliptic flow of charm quarks immediately before hadronization. The impact of the later hadronic phase is shown to play a minor role. One should note that the results are very sensitive to the details of the hadronization mechanism, i.e. on the probability to hadronize through coalescence or through Peterson fragmentation and to the choice of the parameters in each hadronization channel.

Our study confirms that even at low collision energies the charm quarks can be an invaluable tool to probe the properties of the QCD-medium. Nevertheless, there are shortcomings in the present approach: (I) we rely on Pythia with default *SoftQCD* mode settings to produce the initial charm-quark momentum distribution in p–p collisions, but maybe a fine tuning of the settings might produce noticeable differences. Unfortunately, common models and tools like FONLL [87–90] or HERWIG [91], strongly based on pQCD, are not very reliable in this low-energy range. (II) In the FAIR-energy regime, we miss one of the main advantages of studying heavy flavors, i.e. precise pQCD based predictions of the charm-quark initial states, mentioned also in the introduction. (III) In principle the coarse-graining approach would allow us to start the Langevin propagation earlier than in the hydro case, resulting in a clear improvement of the naive assumption of no interaction at all until full thermalization. Moreover, we should also introduce a time delay before the spatial separation of the $c - \bar{c}$ couple after its formation is large enough to be considered a “colored” object. Since the results obtained so far point toward a major role of the early dynamics of the system, it is definitely very important to develop a more realistic treatment of this stage. (IV) The hydro model might be improved by taking into account viscous effects, which are not completely negligible at low collision energies, and possibly anisotropic hydrodynamics, which would allow to slightly anticipate the propagation even in the hydro case. (V) To partially take into account the hadronic interactions, in the version of the UrQMD/hybrid model adopted in this work we stop the simulations at temperatures slightly below $T_c$, when, in principle, the fluid description of the medium should be replaced by a transport model, like in the standard UrQMD/hybrid model. We might improve this situation by restoring the full UrQMD/hybrid approach, but neglecting the back-reactions of the $\text {D}$ mesons on the other particles during their mutual interactions. This strategy would provide a more realistic modeling of the hadronic phase, while preserving the possibility of oversampling the $\text {D}$ mesons, which is an almost essential condition to collect a sufficient statistics in an energy regime quite close to the $c-\bar{c}$ production threshold. (VI) Another very important limitation of our model is the hadronization method. As we discussed in Sect. 6.3, here further improvements of the fragmentation function for low momenta and on the coalescence model are strongly desired. (VII) We limited our study to $\text {D}^{\pm }$, $\text {D}^0$ and $\bar{\text {D}}^0$ mesons, however, in a more comprehensive study, excited states and strange $\text {D}$ mesons should be included as well.

To conclude, the work that we just presented provides useful indications about the direction in which further and more refined studies should focus. Despite their low production rate, the study of the elliptic flow of charmed mesons carries a wealth of information about the QGP and the QCD also in the FAIR energy range.

## Data Availability

This manuscript has no associated data or the data will not be deposited. [Authors’ comment: This manuscript has no associated data.]

## Footnotes

1 We use a non-standard definition of $R_{AA}$ as the ratio between the normalized transverse momentum distribution of $\text {D}$ mesons in ion-ion collisions and the normalized transverse momentum distribution in proton-proton collisions. By this we take out the unknown yields of the $\text {D}/\bar{\text {D}}$ mesons in pp and AA collisions at this low energy. Moreover, we call collectively $\text {D}$ mesons the $\text {D}^+(c\bar{d})$ and the $\text {D}^0(c\bar{u})$, we call $\bar{\text {D}}$ the $\text {D}^-(\bar{c}d)$ and the $\bar{\text {D}}^0(\bar{c}u)$.

2 For technical reasons in Pythia the simulations are performed in a fixed target set-up and the four-momenta are boosted back to the center of mass frame.
